# Consistency of frontal cortex metabolites quantified by magnetic resonance spectroscopy within overlapping small and large voxels

**DOI:** 10.1038/s41598-023-29190-y

**Published:** 2023-02-08

**Authors:** Marilena M. DeMayo, Alexander McGirr, Ben Selby, Frank P. MacMaster, Chantel T. Debert, Ashley D. Harris

**Affiliations:** 1 Department of Radiology, University of Calgary, Calgary, AB Canada; 2 Department of Psychiatry, University of Calgary, Calgary, AB Canada; 3 Hotchkiss Brain Institute, University of Calgary, Calgary, AB Canada; 4 Mathison Centre for Mental Health Research and Education, Calgary, AB Canada; 5 Alberta Children’s Hospital Research Institute, University of Calgary, Calgary, AB Canada; 6 Non-Invasive Brain Stimulation Network (N3), Cumming School of Medicine, University of Calgary, Calgary, AB Canada; 7 Department of Psychiatry, Dalhousie University, Halifax, NS Canada; 8 IWK Health, Halifax, NS Canada; 9 Department of Clinical Neurosciences, University of Calgary, Calgary, AB Canada

**Keywords:** Biological techniques, Neuroscience

## Abstract

Single voxel magnetic resonance spectroscopy (MRS) quantifies metabolites within a specified volume of interest. MRS voxels are constrained to rectangular prism shapes. Therefore, they must define a small voxel contained within the anatomy of interest or include not of interest neighbouring tissue. When studying cortical regions without clearly demarcated boundaries, e.g. the dorsolateral prefrontal cortex (DLPFC), it is unclear how representative a larger voxel is of a smaller volume within it. To determine if a large voxel is representative of a small voxel placed within it, this study quantified total N-Acetylaspartate (tNAA), choline, glutamate, Glx (glutamate and glutamine combined), *myo*-inositol, and creatine in two overlapping MRS voxels in the DLPFC, a large (30×30x30 mm) and small (15×15x15 mm) voxel. Signal-to-noise ratio (SNR) and tissue type factors were specifically investigated. With water-referencing, only *myo*-inositol was significantly correlated between the two voxels, while all metabolites showed significant correlations with creatine-referencing. SNR had a minimal effect on the correspondence between voxels, while tissue type showed substantial influence. This study demonstrates substantial variability of metabolite estimates within the DLPFC. It suggests that when small anatomical structures are of interest, it may be valuable to spend additional acquisition time to obtain specific, localized data.

## Introduction

Single voxel magnetic resonance spectroscopy (MRS) experiments aim to quantify metabolite concentrations within a predefined volume of tissue. As MRS voxels are rectangular prism shapes, either the voxel must be a small volume to be contained within the tissue of interest or it will include neighbouring tissue that is not of interest, i.e., partial volume effects. Voxel placement is particularly relevant when investigating cortical regions, where anatomical boundaries demarcating regions of different physiological significance are not clear, such as the dorsolateral prefrontal cortex (DLPFC).

In MRS, the voxel is often larger than the structure or tissue of interest both to ensure it encompasses the tissue of interest and to ensure adequate signal to noise ratio (SNR). A larger voxel is often needed in regions with poorly defined anatomical boundaries, where inter-individual and operator variability might lead to incomplete or inaccurate sampling^1^. Moreover, there is a significant SNR benefit to a larger voxel as compared to a small voxel; SNR is proportional to the volume of the voxel and the square root of the number of averages^2^. Thus, for a 50% reduction in volume, four times the number of averages is needed for a comparable SNR. Therefore, from a SNR perspective, it is more efficient to increase voxel size rather than increase number of averages, especially as increasing the number of averages extends the scan time, creating an additional risk of motion. It is, however, unclear the degree to which partial volume effects impact metabolite measurements. In MRS, it is generally accepted that voxel location has a significant effect on metabolite levels. To our knowledge, the degree to which increasing the voxel size, and incorporating more tissue, possibly outside the volume of interest, compromises the regional sensitivity of the measure has not been specifically examined. Characterising this regional variability is valuable, as if there is little regional variability, larger voxels could be used to acquire comparable information with higher SNR and shorter acquisition time.

To address the question "is a larger, more inclusive, cortical voxel representative of a smaller, more anatomically specific voxel?”, this study investigated two overlapping MRS voxels placed within the right DLPFC. One voxel was large, thus subject to greater partial volume effects, whereas the other was small, and required significantly more averages to achieve comparable SNR. The large voxel was 27 cubic centimetres and, while larger than typical for PRESS voxels, is common for MEGA-PRESS and thus is often used in PRESS when matching with an edited acquisition^3–6^. The small voxel was 3.38 cubic centimetres, in order to capture a more anatomically precise region of the DLPFC. The relationship between metabolite levels (both concentration and creatine-referenced) within the small and large voxels was quantified to understand regional variability within the DLPFC.

## Methods

This study was approved by the University of Calgary Institutional Review Board (REB18-1976) and all participants provided informed consent. This study was performed in accordance with relevant guidelines and regulations.

### Acquisition

**Figure 1:**
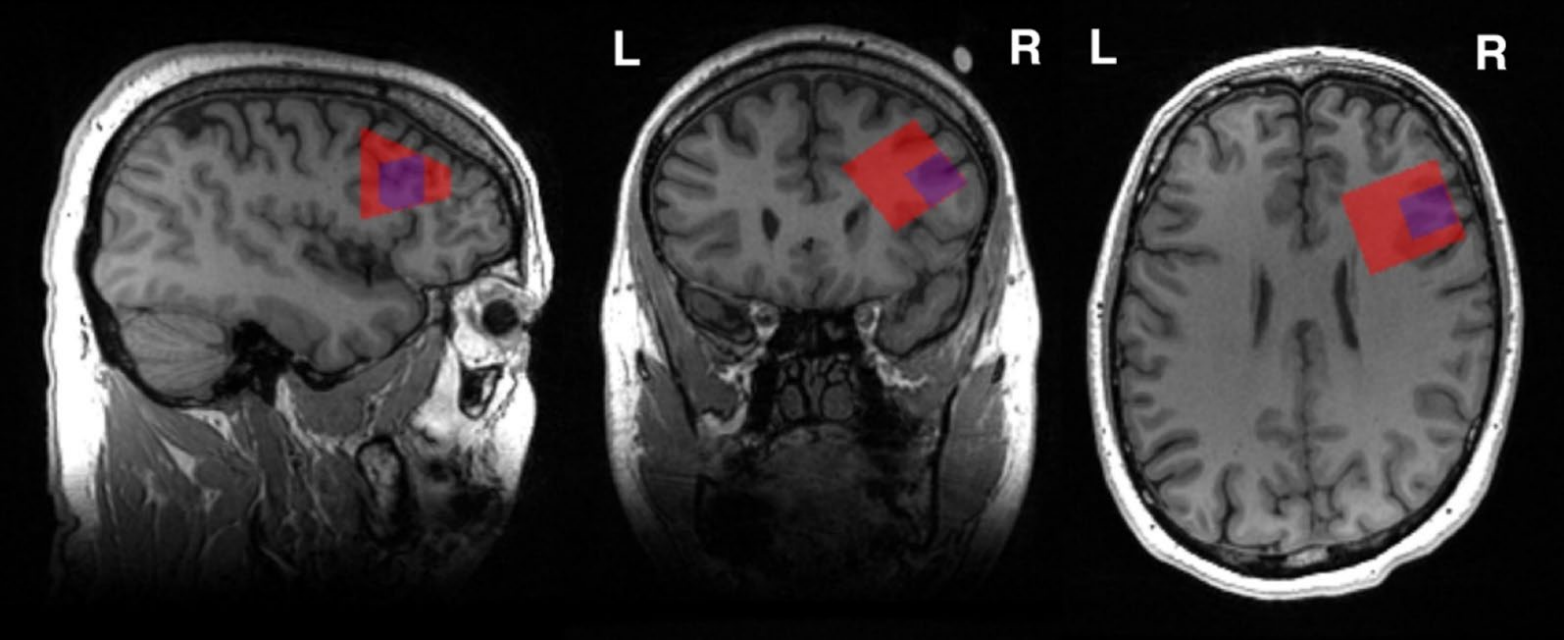
An example voxel placement of the large, red, and small, purple, voxels in the right DLPFC.

Data were acquired from 18 participants, aged 28–65 years (M = 51.39, SD = 10.39), 15 males). Scanning was conducted on a 3 T GE 750W scanner using a 32-channel head coil and running software version DV25.0_R02_1549.b. Data was collected by one co-author, BS. As is our standard procedure, padding around the head of the participant was used to minimize the possibility of motion while trying to maximise participant comfort. A T1-weighted fast spoiled gradient recalled echo (FSPGR) brain volume (BRAVO) anatomical image (TR = 7.26 ms, TE = 2.656 ms, FA = 10°, voxel size = 0.9375 × 0.9375 × 1 mm) was acquired for voxel placement and tissue segmentation. Two short-echo Point REsolved SpectroScopy (PRESS) acquisitions^7^ using the GE provided PRESS with CHEmical Shift Selective saturation (CHESS) water suppression were acquired in the DLPFC. For both MRS acquisitions, the parameters were: TR = 1800 ms, TE = 35 ms, 4096 points, 5 kHz bandwidth, two-step phase cycle. Linear first order shims were applied as part of the GE auto prescan process. The small voxel was 15 × 15x15 mm^3^ with 200 water-suppressed averages and 16 water-unsuppressed averages, taking approximately 6.5 min. The large voxel was 30 × 30x30 mm^3^, with 64 water-suppressed averages and 16 water-unsuppressed averages, taking approximately 2.5 min. For localization of the right DLPFC, Vitamin E capsules were placed prior to scanning according to the Beam-F4 method^8^, which locates the DLPFC using skull surface anatomy. This was used to inform the initial placement of the voxel, with the T1-weighted image used to centre the voxel on the nearest gyrus to the capsule, and positioned to avoid any inclusion of the dura. An illustrative voxel placement of both the large and small voxels is shown in Fig. 1.

### Anatomical registration

The voxels were coregistered to the T1-weighted image and segmented using the Gannet CoRegStandAlone function^9^, which calls SPM12^10^ to estimate the voxel fraction of grey matter (GM), white matter (WM) and cerebrospinal fluid (CSF). To check voxel overlap within participants, as in to quantify the overlap between the small voxel and the large voxel for each individual, the masks of the two voxels were overlaid and the dice coefficient was calculated based on how much of the small voxel was within the large voxel^1, 11^.

### MRS processing

Data were pre-processed using the automated FID-A pipeline, which includes coil combination, removal of bad averages and spectral registration^12^, and metabolites were quantified using LCModel^13^. Within our pipeline, averages (individual free induction decays) were automatically removed. Basis sets for quantification were simulated using the FID-A toolbox^12^ based on sequence timing and RF pulse shape. The basis set included: alanine, aspartate, glycerophosphocholine, phosphocholine, creatine, phosphocreatine, gamma-aminobutyric acid (GABA), glutamate, glutamine, lactate, inositol, N-acetyl aspartate (NAA), N-acetylaspartylglutamate (NAAG), scyllo-inositol, glutathione, glucose, and taurine, with the default macromolecule simulation parameters from LCModel. Reported SNR and linewidth is from FID-A. Data quality was visually assessed by two raters and metabolites with a Cramer-Rao Lower Bounds of greater than 20% or with values that were greater than three standard deviations from the mean were excluded.

The metabolites of interest in this study were based on those reliably quantified from a standard PRESS research scan: tNAA (NAA + NAAG), choline (from glycerophosphocholine + phosphocholine), glutamate, Glx (glutamate + glutamine), *myo*-inositol, and creatine (from creatine + phosphocreatine) with the default macromolecule simulation parameters from LCModel. Water-referenced metabolite data were tissue corrected to millimolal values, accounting for tissue specific T1- and T2- water and metabolite relaxation, proton density and CSF fraction, as per Gasparovic, Song^14^. Full details are included in the supplementary information and the script used to do this is available at https://github.com/HarrisBrainLab/TissueCorrections. Metabolite levels were also quantified using creatine-referencing in a secondary analysis.

The smaller voxel was expected to have a lower SNR than the large voxel, even with the longer acquisition. To investigate the contribution of SNR to the metabolite correspondence between voxels, to match a standalone acquisition we used the first 8 water-suppressed and first 8 water-unsuppressed averages of the large voxel to generate a “large voxel with matched SNR” to match the SNR of the small voxel. Processing of this data was as above (pre-processed, quantified and tissue corrected using the same routine). This is referred to as the “large voxel with matched SNR” throughout.

### Analysis

Metabolite levels between the small voxel and the large voxel with full SNR were compared with paired *t*-tests. Metabolite correspondence was assessed using Spearman’s *rho*, chosen to reflect the differences in distributions between the voxels. To examine the correspondence of the large voxel with full SNR to the small voxel, metabolite values were correlated, using both the water-referencing (as per consensus recommendations^15–17^) and creatine-referencing (to investigate the effect of the water signal). In order to investigate the impact of SNR on the correspondence between voxel, this analysis was repeated with metabolite values from the large voxel with matched SNR correlated to the small voxel, using both water and creatine-refencing. Bland–Altman plots were constructed using the Rik^18^ toolbox in MATLAB to assess agreement and for the presence of bias of measurement between the small voxel and the large voxel with full SNR. To evaluate the association between metabolite levels and tissue composition, correlations with Spearman’s *rho* were conducted between GM fraction and each metabolite, separately within large voxel with full SNR and small voxel.

## Results

### MRS processing

**Figure 2:**
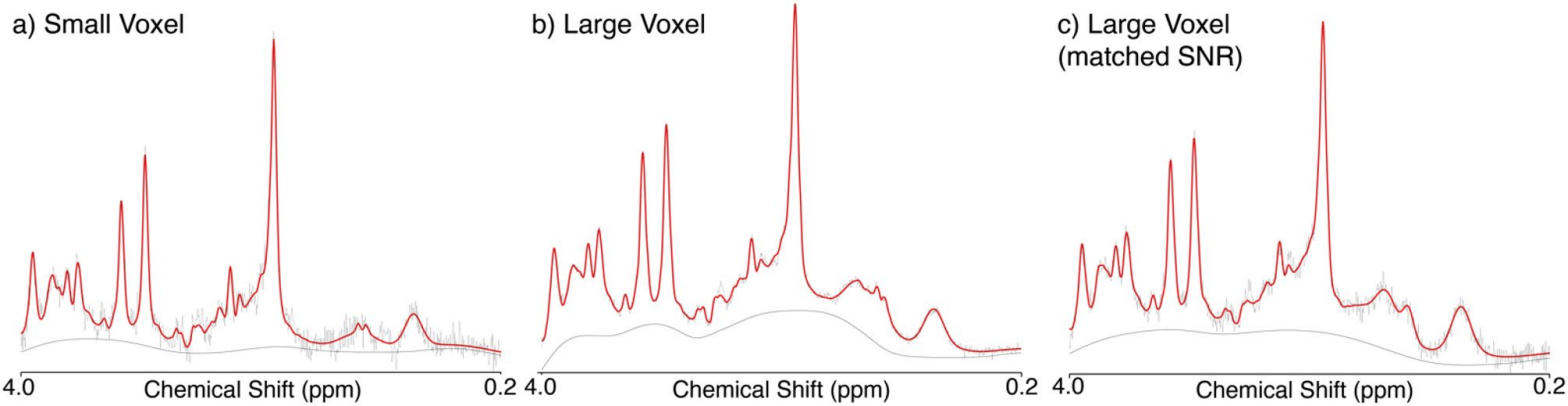
Exemplar spectra from one participant for the (**a**) small voxel, (**b**) large voxel with full signal, and (**c**) large voxel with reduced signal.

**Figure 3:**
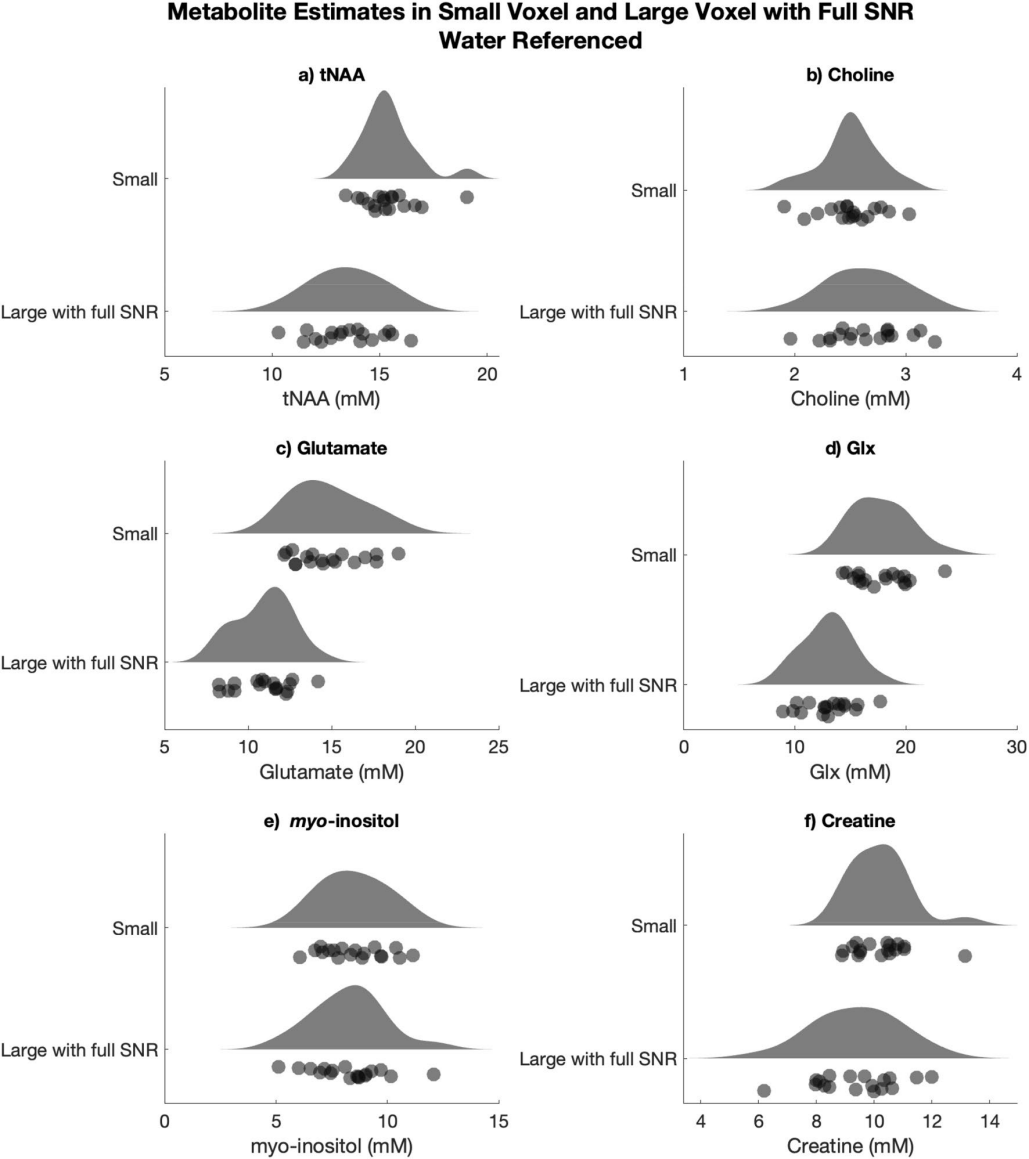
Raincloud plots of the milli-molal metabolite values of the small voxel (top) and full SNR large voxel (bottom). Raw data values are displayed below the probability distribution: a histogram smoothed with a kernel of 1 mm. Developed using^19^.

All data passed visual inspection and metrics for data inclusion, with no metabolite values greater than three standard deviations from the mean. From the large voxel with full SNR (which has the shorter acquisition time) across participants, typically no FIDs were removed (15 participants); in two participants 1 average was removed and in one participant 2 averages were removed by the FID-A preprocessing^12^. For the large voxel with matched SNR, none of the included averages (the first 8) were removed. In the FID-A preprocessing^12^ for the small voxel with the longer acquisition, no averages were removed for 6 participants, a mean of 2.4 averages and median of 2 averages were removed, with a range of 0 to 9. Example spectra with the LCModel fit are shown in Fig. 2. The data and distributions of metabolite estimates from the large voxel and small voxel are detailed in Table 1 and shown in Fig. 3.

**Table 1:** Metabolite levels (milli-molal), metabolite Cramer-Rao Lower Bounds (CRLBs) and tissue fraction of the gray matter (GM), white matter (WM), cerebrospinal fluid (CSF) and GM fraction (GM/[GM + WM]) for the small and full SNR large voxel. Values are means with standard deviations in brackets. The results of two-tailed paired t-test comparing differences between voxels.

	Small voxel	Large voxel with full SNR	Comparison
tNAA (mM)	15.42 (1.27)	13.50 (1.63)	*t*(17) = 4.21, *p* < 0.0001
Choline (mM)	2.50 (0.27)	2.64 (0.34)	*t*(17) = -1.62, *p* = 0.12
Glutamate (mM)	14.80 (2.07)	10.95 (1.66)	*t*(17) = 7.03, *p* < 0.0001
Glx (mM)	17.73 (2.45)	12.98 (2.23)	*t*(17) = 6.85, *p* < 0.0001
myo-inositol (mM)	8.52 (1.44)	8.25 (1.62)	*t*(17) = 0.76, *p* = 0.46
Creatine (mM)	10.21 (1.02)	9.38 (1.44)	*t*(17) = 2.67, *p* = 0.016
tNAA (CRLB)	3.11 (0.68)	2.39 (0.61)	*t*(17) = 4.58 *p* < 0.001
Choline (CRLB)	4.50 (0.86)	2.78 (0.65)	*t*(17) = 8.17, *p* < 0.0001
Glutamate (CRLB)	9.22 (2.37)	6.44 (1.72)	*t*(17) = 6.12, *p* < 0.0001
Glx (CRLB)	8.67 (1.82)	7.56 (1.79)	*t*(17) = 4.75, *p* = 0.026
myo-inositol (CRLB)	7.17 (1.82)	4.39 (0.70)	*t*(17) = 6.43, *p* < 0.0001
Creatine (CRLB)	3.72 (0.83)	2.33 (0.49)	*t*(17) = 8.44, *p* < 0.0001
GM	45.87% (9.61)	35.55% (3.56)	*t*(17) = 4.75, *p* < 0.0001
WM	45.40% (13.40)	56.90% (5.33)	*t*(17) = -4.08, *p* < 0.0001
CSF	8.73% (5.25)	7.55% (3.32)	*t*(17) = 1.37, *p* = 0.19
GM fraction	50.77% (12.70)	38.52% (4.31)	*t*(17) = 4.38, *p* < 0.0001

Quality metrics for the two voxels are summarised in Table 2. The measured SNR was significantly different between the small voxel and large voxel with full SNR, *t*(17) = 20.23, *p* < 0.0001. For the large voxel with matched SNR, and the small voxel, there was no significant difference in SNR, *t*(17) = 0.46, *p* = 0.65. The linewidth was significantly different between both the full SNR and small voxel, *t*(17) = 4.30, *p* < 0.0001, and the large voxel with matched SNR and small voxel, *t*(17) = 7.29, *p* < 0.0001.

**Table 2:** Quality metrics of the voxels. Signal to Noise Ratio (SNR) calculated with the height of the tNAA peak to the signal at 0 to -2 ppm. Linewidth is the width of the water peak in Hz. Both calculated using FID-A^12^. Values are means with standard deviations in brackets.

	Small voxel	Large voxel full SNR	Large voxel with matched SNR	Small vs. large with full SNR	Small vs. large with matched SNR large
SNR	38.15 (6.53)	75.45 (34.87)	38.82 (7.96)	*t*(17) = 5.10, *p* < 0.0001	*t*(17) = 0.43, *p* = 0.67
Linewidth (Hz)	8.14 (0.65)	12.33 (2.41)	12.29 (2.44)	*t*(17) = 8.13,*p* < 0.0001	*t*(17) = 7.91, *p* < 0.0001

### Voxel anatomy

**Figure 4:**
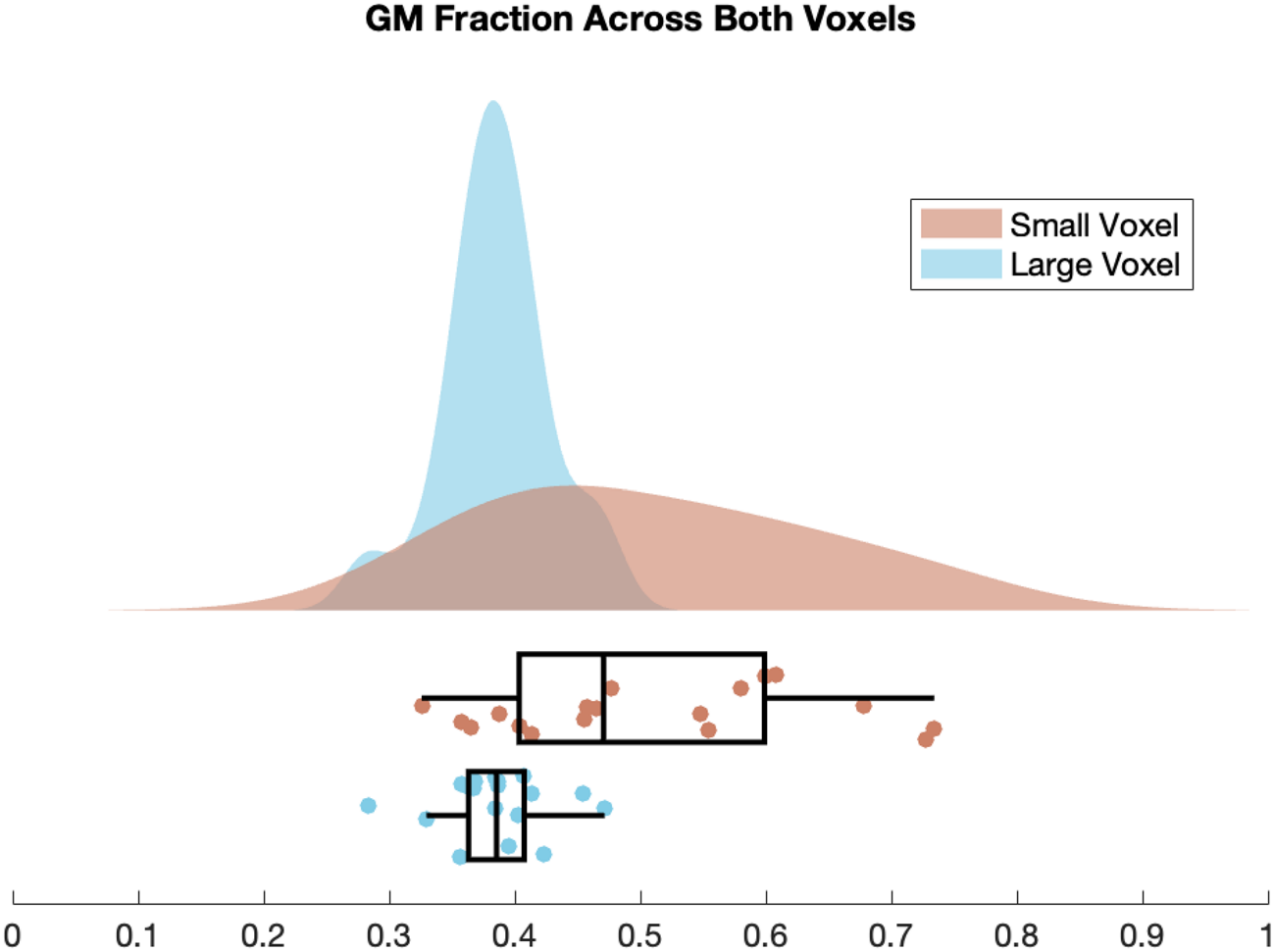
Raincloud plot of GM fraction as proportion of total tissue in the small voxel (light pink, top box plot) and large voxel (blue, bottom box plot). GM fraction calculated as GM/(GM + WM). Raw data values are displayed below the probability distribution: a histogram smoothed with a kernel of 1 mm. The boxplot illustrates the interquartile range with the whiskers being 1.5 × interquartile range. Developed using^19^.

The average voxel overlap was 91.74% (SD = 8.31%). Lower overlap occurred when the smaller voxel was closer to the cortical surface, which was not possible with the larger voxel. The small voxel had significantly greater proportion of GM than the large voxel, t(17) = 4.38, *p* < 0.0001. Proportion of GM, WM and CSF as a percentage within each voxel is reported in Table 1 and GM fraction (GM/total tissue) in each voxel (small and large) is illustrated in Fig. 4.

### Metabolite correspondence

**Figure 5:**
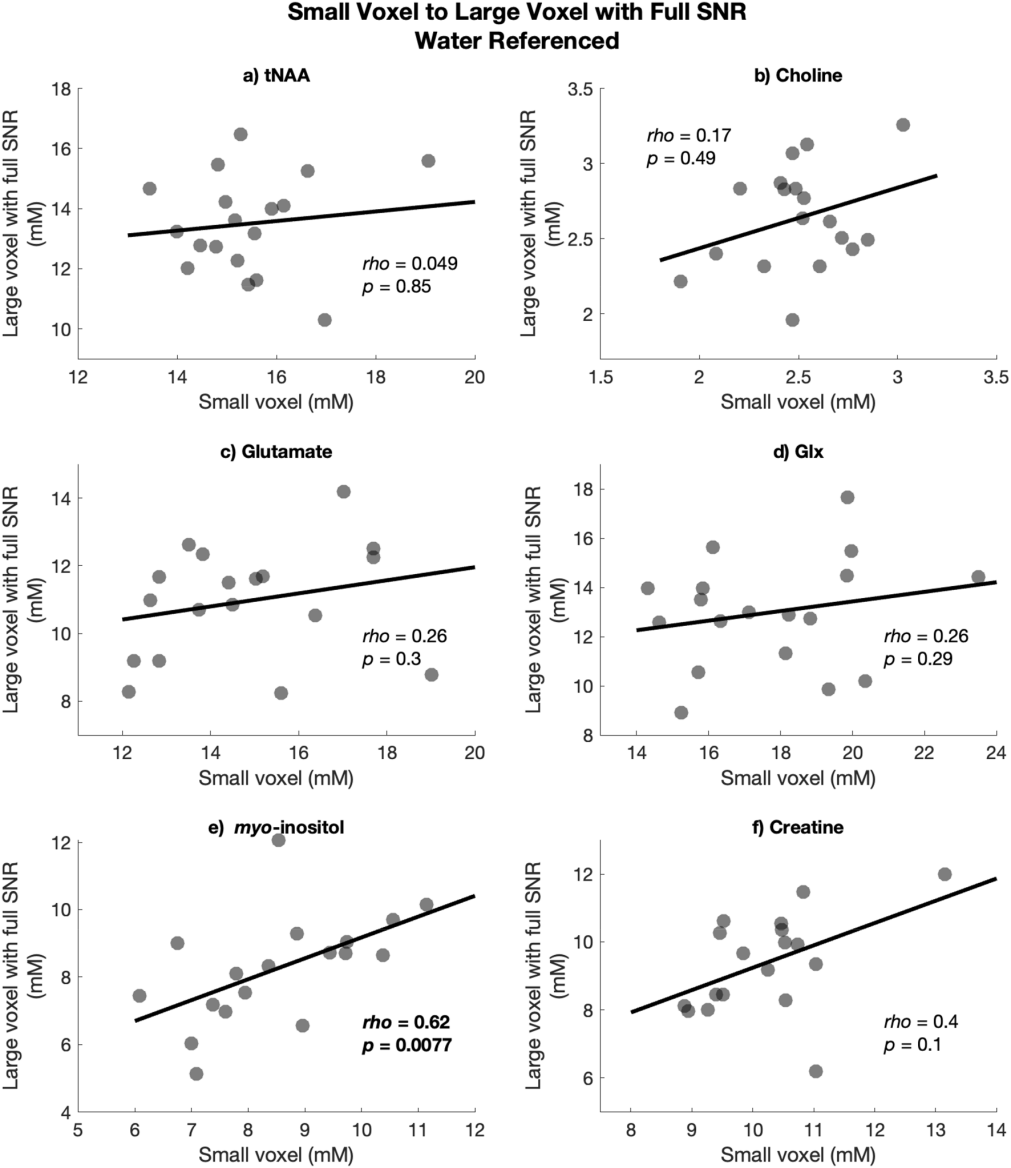
Spearman’s rho correlations between water-referenced (milli-molal) values in the large voxel with full SNR on the y-axis and the small voxel on the x-axis (**a**) tNAA, (**b**) Choline, (**c**) Glutamate, (**d**) Glx, (**e**) myo-inositol and (**f**) Creatine*.*

Only *myo*-inositol was significantly correlated between the large voxel with full SNR and the small voxel (*rho* = 0.62, *p* = 0.0077); no other metabolite showed a significant association between the small and large voxel with full SNR. These associations are illustrated in Fig. 5.

**Figure 6:**
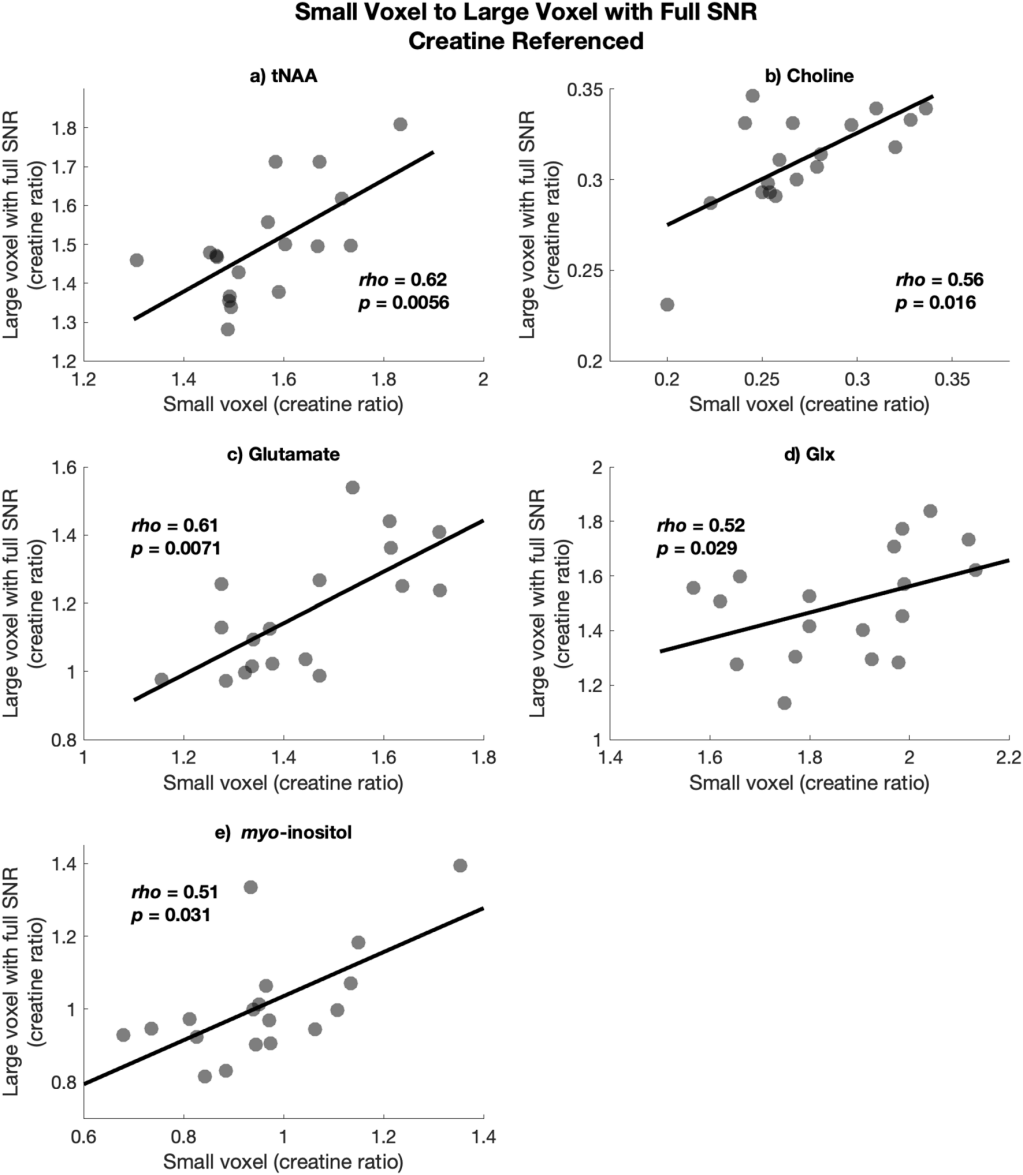
Spearman’s rho correlations between creatine-referenced values in the large voxel with full SNR on the y-axis and the small voxel on the x-axis for (**a**) tNAA, (**b**) Choline, (**c**) Glutamate, (**d**) Glx, and (**e**) myo-inositol.

Repeating this analysis with the creatine referenced values, all metabolites showed a significant correlation between the small voxel and large voxel with full SNR (all *p* < 0.05), illustrated in Fig. 6.

**Figure 7:**
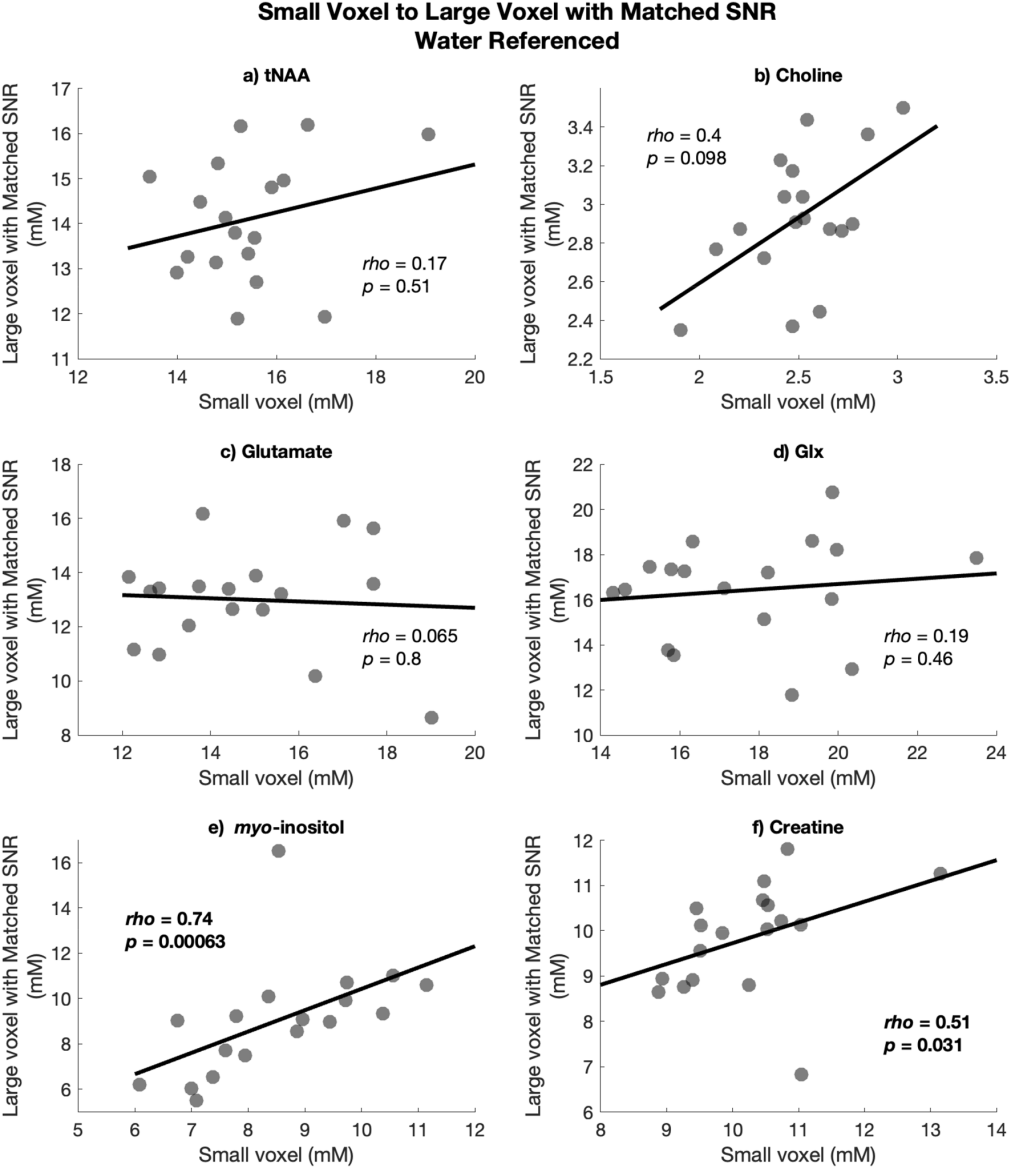
Spearman’s rho correlations between water-referenced (milli-molal) values in the large voxel with matched SNR on the y-axis and the small voxel on the x-axis for (**a**) tNAA, (**b**) Choline, (c) Glutamate, (**d**) Glx, (**e**) myo-inositol and (**f**) Creatine.

**Figure 8:**
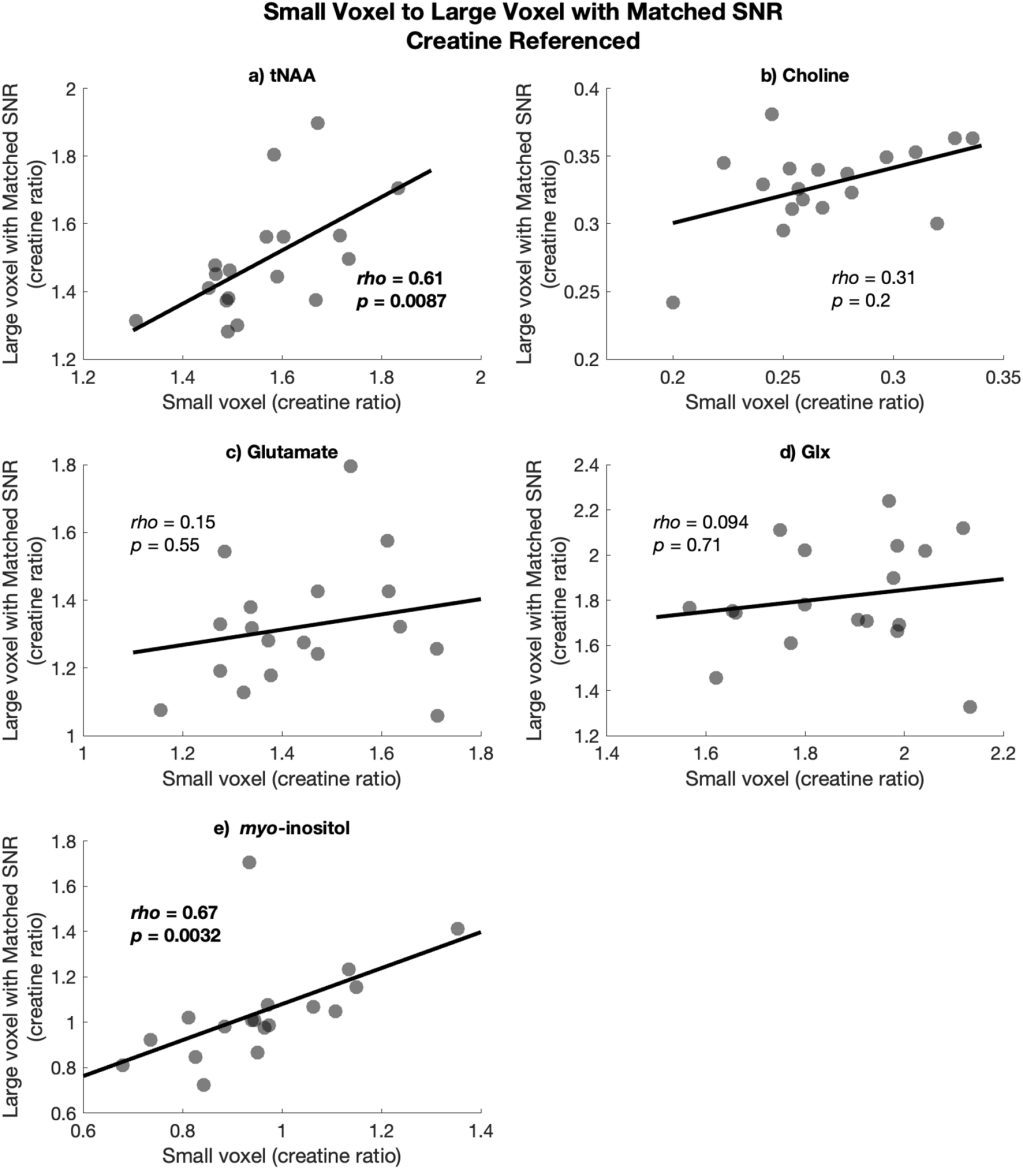
Spearman’s rho correlations between creatine-referenced values in the large voxel with matched SNR on the y-axis and the small voxel on the x-axis for (**a**) tNAA, (**b**) Choline, (**c**) Glutamate, (**d**) Glx, and (**e**) myo-inositol.

The correlational analyses were then performed using the metabolite values from the large voxel with matched SNR and the small voxel. With water referencing, there were significant associations between *myo*-inositol (*rho* = 0.74, *p* < 0.001) and creatine (*rho* = 0.51, *p* = 0.031) in the two voxels. This is illustrated in Fig. 7. With creatine referencing, the association between *myo*-inositol remained significant (*rho* = 0.67, *p* = 0.0063) and tNAA became significant (*rho* = 0.61, *p* = 0.0087). The other metabolites did not show a significant association (all *p* > 0.2). These analyses are shown in Fig. 8.

### Bland Altman plots

**Figure 9:**
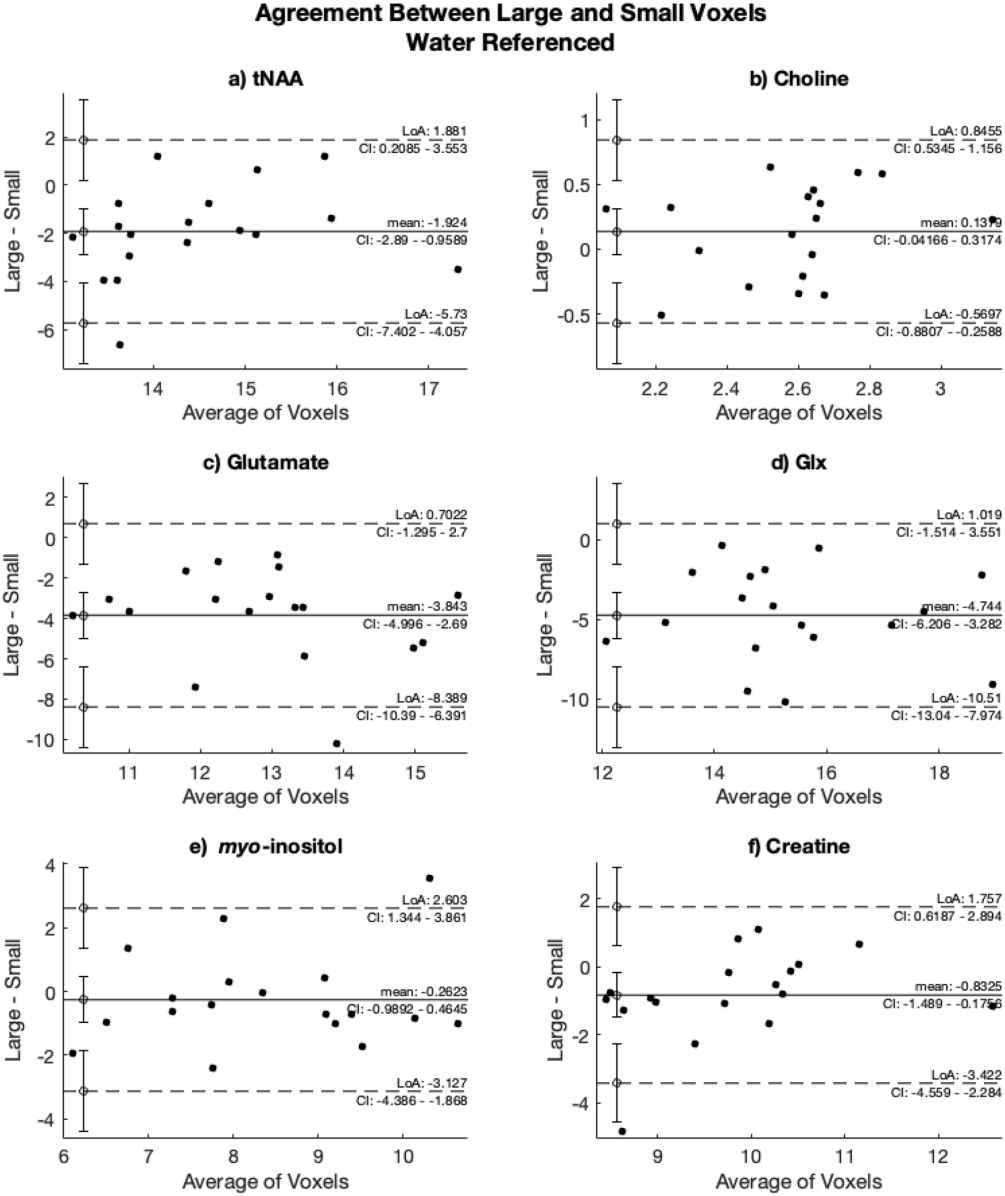
Bland–Altman plots illustrating agreement between the large voxel with full SNR and the small voxel, using the water-referenced data for (**a**) tNAA, (**b**) Choline, (**c**) Glutamate, (**d**) Glx, (**e**) myo-inositol and (**f**) Creatine. The y-axis shows the difference between the large and small voxels (large minus small) and the x-axis shows the average of the metabolite estimate.

Bland–Altman plots assessing agreement between data from the large voxel with full SNR and the small voxel are shown in Fig. 9. There was a proportional bias for creatine and tNAA; as the measured metabolite levels increased, there was a greater difference between the two voxels with the large voxel showing higher metabolite levels than in the small voxel. None of the plots evidenced a systematic bias.

### Tissue contribution

**Figure 10:**
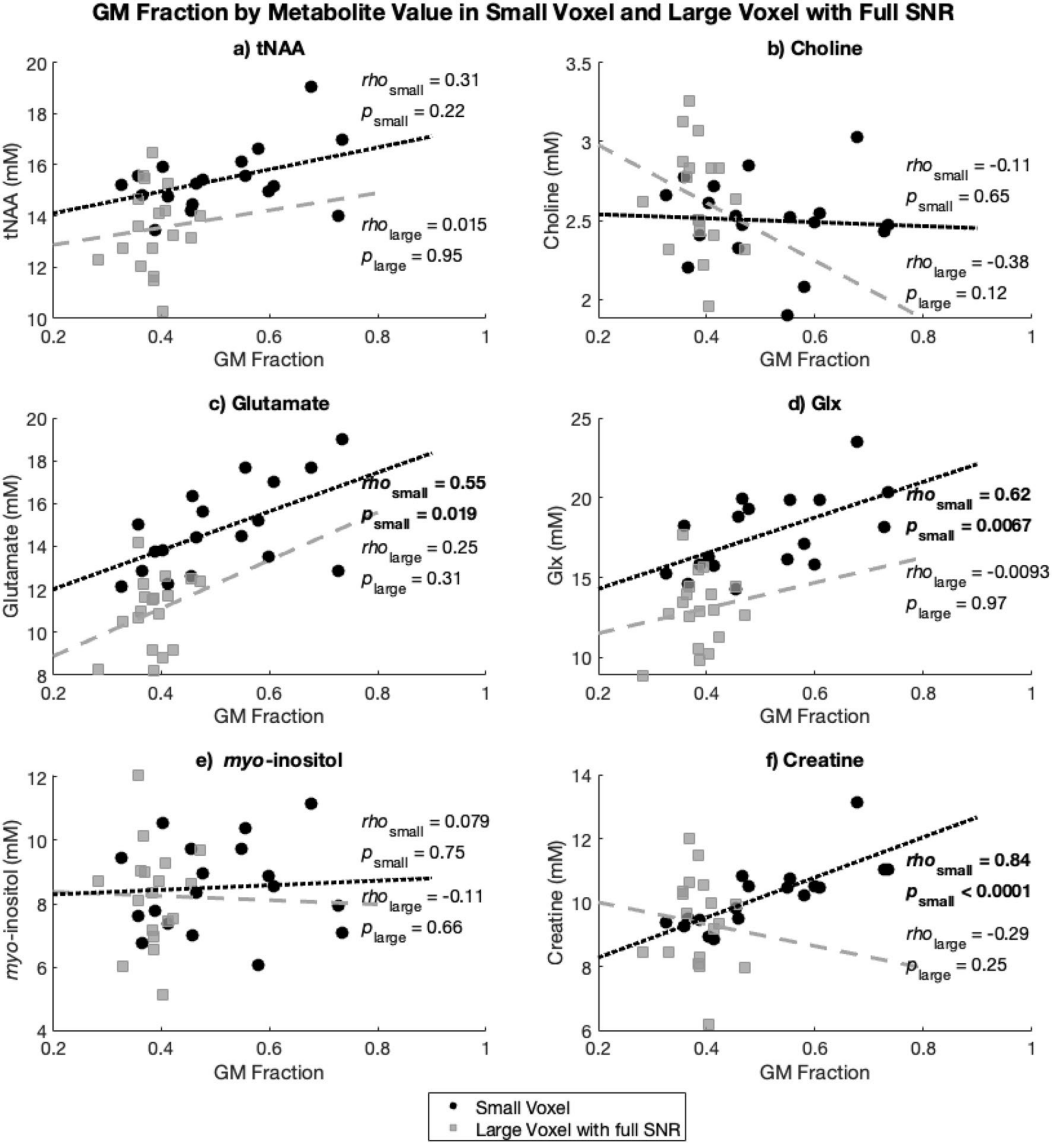
GM fraction by metabolite molal value for the small voxel (circles, with dotted line) and the large voxel with full SNR (squares, dashed line) for (**a**) tNAA, (**b**) Choline, (**c**) Glutamate, (**d**) Glx, (**e**) myo-inositol and (**f**) Creatine.

To evaluate the association between metabolite levels and tissue composition, correlations with Spearman’s *rho* were conducted between GM fraction and each metabolite, separately within the the large voxel with full SNR and small voxel. Significant associations between GM fraction and metabolites were seen in the small voxel for glutamate (*r* = 0.55, *p* = 0.019, Glx (*r* = 0.62, *p* = 0.0067) and creatine (*r* = 0.84, *p* < 0.0001). There were no associations between GM fraction and metabolites in the large voxel. The correlations between GM fraction and metabolite levels are illustrated in Fig. 10.

## Discussion

This study compared metabolite levels measured in a small voxel encompassed in a large voxel to understand how well a large voxel, with much greater SNR yet compromised by partial volume effects, can approximate metabolite levels in a smaller region of interest. The small voxel was 3.38 cubic centimetres, which, while small, is not unprecedented^17–23^. The small size of the voxel motivated a longer acquisition of 200 averages, which is more than typically used for standard PRESS acquisitions. The large voxel was 27 cubic centimetres and, while larger than typical for PRESS voxels, is common for MEGA-PRESS and thus is often used in PRESS when matching with an edited acquisition^3–6^. Given the large voxel volume, few averages are required for high quality data. While as few as 8 averages can result in matched SNR, acquiring 64 averages has a low acquisition time burden. Other acquisition parameters (e.g., TE, TR) were consistent between the two acquisitions and with what is commonly used and recommended in the literature^15^.

Overall, water-referenced metabolite levels between the two voxels did not correlate, with the exception of *myo*-inositol. Given the small voxel was encompassed by the larger voxel, this suggests a high degree of regional specificity in metabolite concentrations. It is notable that *myo*-inositol, an osmolyte^24^, was the only metabolite consistently correlated between the two voxels and this correlation was maintained with both creatine-referencing and when the SNR of the large voxel was matched to that of the small voxel. The high correlation of *myo*-inositol between the two voxels suggests greater homogeneity (including smaller differences in concentration between WM and GM, discussed below) of *myo*-inositol across the brain compared to the other metabolites. In the primary analysis, SNR was significantly different between the two voxels, even with the increased number of averages in the small voxel. To examine the impact of SNR on correspondence between the voxels, the first 8 averages of the large voxel were processed to generate data for a large voxel with matched SNR. When SNR was matched and metabolites were referenced to water, the *myo*-inositol correlation between the two voxels remained significant and creatine levels between the two voxels were significantly correlated. The lack of correlation between other metabolites with the matched SNR, water-referenced, analysis suggests that the general pattern of results with water referencing was not driven by SNR differences between the voxels. Related to SNR are the significant differences in linewidth between the large and small voxels. A better shim, resulting in a more homogenous field, is more easily achieved for the small voxel and it is worth noting that the scanner (a GE 750w) only has first order shims. This better shim in the small voxel would associate with narrower line width seen in the small voxel and likely results in better measurement precision for metabolites. However, the lack of precision of the larger voxel indicated by differences in linewidth is random, so does not explain the lack of correspondence between voxels.

To investigate whether the water signal systematically affected the results, all analyses were repeated with creatine referencing. Creatine is proposed to act as an internal tissue control^25^, though water referencing is recommended practice^16^. The full SNR creatine-referenced metabolite concentrations showed a significant association between the large and small voxels for all the metabolites. This suggests that lack of correspondence in the water referenced analysis may be a result of water signal variation between the two voxels, which in turn impacts metabolite quantification. We did also compare transmit gain values to examine whether the resultant power differed between the small and large voxels, and explained variation in water signal between the two voxels. There was no difference between the voxels in transmit gain, nor did it relate to metabolite signal. The correlations between creatine referenced metabolite values were impacted by data quality, as the correlations with large voxel full SNR are not fully replicated in the degraded SNR analysis. We propose the high SNR of tNAA enabled the maintenance of the relationship between the large and small voxel measures but for the other metabolites with lower SNR, the signal variability is too great to evidence correspondence between measures.

A factor worthy of discussion is of chemical shift displacement effects and how these may impact the results of this study. In this implementation of PRESS, the encoding frequency was 2.3 ppm, so creatine and tNAA would have experienced less displacement than *myo*-inositol, glutamate (and glx), water and even choline. While there are research sites that realign voxel location prospectively to account for chemical shift displacement effects, this study used a scanner without this capacity, as is common on clinical systems. As there is greater chemical shift displacement with increasing voxel size^17^, the chemical shift displacement effects would be greater in the large voxel than the small voxel. Given the smaller voxel was generally placed within the larger voxel, the majority of metabolite volumes would still be contained within the larger voxel, even with chemical shift displacement effects. Furthermore, chemical shift displacement effects alone do not explain the lack of correlation between the two voxels, as *myo*-inositol (at 3.56 ppm, thus more affected than choline) consistently showed a strong correlation between the large and small voxel. Nonetheless, chemical shift displacement effects may compound variability introduced by differences in voxel tissue, particularly for the water referenced data.

There are multiple challenges related to the size of MRS voxels in single-voxel spectroscopy. First, there were substantial differences in tissue composition between the two voxels, both in tissue content (as in GM and WM proportions) and cortical structure. The smaller voxel was often positioned closer to the cortical surface to best represent the DLPFC, incorporating proportionally more GM and more variable cytoarchitecture, cell types and density^26^. While MRI cannot measure subtle differences in cortical structure, differences in bulk tissue (GM/WM) can be measured and impact MRS measurements of metabolites. There were no correlations between GM fraction and metabolite concentrations in the large voxel, though this likely reflects the small range of GM fraction values. As expected, the small voxel showed greater variability in voxel tissue fraction because it is more susceptible to voxel placement and composition. In the small voxel were significant positive correlations between GM fraction and glutamate, Glx, and creatine. This is consistent with previous literature in which creatine^27–30^, glutamate^31, 32^ and Glx^32, 33^ levels have been shown to be higher in GM than WM. Differences in metabolite levels between tissue types, seen both in our correlations with GM and in literature, may contribute to the lack of correlation between voxels for these metabolites. tNAA, choline and *myo*-inositol levels did not show correlations with GM fraction, suggesting that for these metabolites tissue type is less likely to be driving differences between voxels. Typically, choline is higher in WM^27, 29, 33^, coherent with our negative *rho* values (though not statistically significant here). It may be that the small sample size is not reflecting this choline-tissue relationship. For tNAA, the literature is mixed: with evidence for higher levels of tNAA in GM^27,30^, higher levels of tNAA in WM^29, 33^ and no differences between tissue types for NAA (not tNAA; 31). Differences between NAA and NAAG may be contributing to this variability^34^; NAA appears to be higher in GM while NAAG is greater in WM^35^. There was not a significant relationship between tNAA and GM fraction in this study. Our study showed no relationship between *myo*-inositol and GM fraction, which is consistent with previous findings^28, 31, 33^ and likely contributes to the correlation of *myo*-inositol between the two voxels. It is possible that developing and applying correction factors for the differences in metabolites between WM and GM^36^, as has been developed for GABA^9^, may increase the correspondence between voxels.

Secondly, deciding on voxel shape is a study-specific question for single voxel MRS experiments. For this study, as is common in many MRS studies, we opted for the cuboidal shape as an intermediate solution for measurement within the DLPFC. A rectangular prism would have provided a different representation. Rectangular prism shapes could have provided an option to better match tissue composition between the two voxels. Such prisms, however, may not represent the tissue of interest as closely as a cube. If a rectangular prism was placed with the long edge along the surface of the brain, then it would have to be pulled deeper into the brain to avoid dura and skull. While a rectangular prism with the short edge at the cortical surface would increase the proportion of white matter within the voxel. The solution of shape needs to be tailored to the question of interest. Here, the DLPFC is chosen as an exemplar cortical region, and while our analyses and conclusions may be generalizable, they must be replicated in other regions before concluding a universal phenomenon. The concordance of a small and large voxel will depend on anatomical differences, as well as cellular composition and cytoarchitectonic differences between brain regions and surrounding parenchyma. Further, while technical factors such as shim and SNR were not significant in this DLPFC voxel, they may have differential impacts in other brain regions.

Thirdly, the larger voxel encompasses greater tissue heterogeneity. This may explain the difference in spectral baseline between the small and large voxels. In the large voxel, at 2.5ppm there appears to be an increased macromolecular signal and at 1.3 ppm there appears to be lipid contamination. This lipid contamination may be a result of greater chemical shift displacement effects in the large voxel. We did conduct the analysis with the default basis macromolecular fitting in LCModel, however, these factors still may contribute to differences in tNAA and glutamate/Glx levels between the two voxels.

Overall, there was no predominant systematic bias in metabolite concentrations between the two voxels. For tNAA and creatine, however, there was a subtle proportional bias. Measured tNAA was higher in the small voxel compared to the large voxel at low concentrations, but shifted to more tNAA in the larger voxel at higher concentrations. Tissue composition, including effects of NAA and NAAG signal contributions, may contribute to this proportional bias. As the small voxel could be placed more cortically, it captured proportionally more GM or more dense neuronal tissue, and therefore more tNAA than large voxels positioned further from the cortical surface.

In conclusion, our data shows that there are sufficient regional differences in metabolite concentrations that a large voxel does not represent metabolite concentrations in a small voxel. This is at least partially driven by difference in voxel tissue (GM/WM) composition. Our findings suggest that when interested in a small, anatomically precise region of interest, it is valuable to spend additional acquisition time to obtain specific, localised data using a small voxel; however, this comes with a greater risk of patient movement. We do recognize that some studies are interested in a more representative metabolite measurement and are less concerned about anatomical specificity. Furthermore, a large voxel is typically required when using editing (e.g., to measure GABA or glutathione), in order to obtain adequate signal^37, 38^. This raises a challenging study design issue when researchers are interested in metabolites that require editing and as well as those measured with typical acquisitions such as PRESS; should the PRESS acquisition be matched to the edited acquisition, or should a more anatomically specific PRESS acquisition be used? The decision will depend on the study. More broadly, this study highlights the importance of anatomical specificity (both in voxel size and placement) when conducting MRS experiments and interpreting the literature, particularly when considering studies with similar but slightly different voxel prescriptions.

## Supplementary Information

Supplementary Information 1.

Supplementary Information 2.

## Data Availability

Supplementary Information 2 contains the processed MRS values used for analysis. The datasets generated during the current study are available from the corresponding author on reasonable request.
