# Supplementary material for: Consistency of frontal cortex metabolites quantified by magnetic resonance spectroscopy within overlapping small and large voxels
Source: Sci Rep. 2023 Feb 8;13:2246. doi: 10.1038/s41598-023-29190-y (PMC9908968; doi:10.1038/s41598-023-29190-y)
Supplement: Supplementary file 1 — Supplementary Information 1. [file 41598_2023_29190_MOESM1_ESM.docx]

Tissue correction conducted for molal values as per Gasparovic, et al. (2006, 2018):

$$\left[ M \right]_{\mathrm{molal}}=\frac{(f_{\mathrm{GM}}\times R_{H2O\_\mathrm{GM}}+ f_{\mathrm{WM}}\times R_{H2O\_\mathrm{WM}} + f_{\mathrm{CSF}}\times R_{H2O\_\mathrm{CSF}})}{\left( 1-f_{\mathrm{CSF}} \right) \times R_{M}}\times\frac{S_{M\_obs}}{S_{H2O\_\mathrm{obs}}} x \frac{2}{{\#H}_{M}}\times{[H_{2}O]}_{\mathrm{mola}l}$$

Where

$S_{M\_obs}$ is the observed signal for the metabolite

$S_{H2O\_obs}$ is the observed signal for water (H2O)

${\#H}_{M}$ is the number of hydrogen protons in the metabolite of interest

${[H_{2}O]}_{\mathrm{mola}l}$ is the molality of water within the voxel

$f_{\mathrm{GM}}, f_{\mathrm{WM}}, f_{\mathrm{CSF}}$are the mole fractions of GM, WM and CSF, respectively, with the calculation detailed below

$R_{H2O\_GM} , R_{H2O\_WM} , R_{H2O\_\mathrm{CSF}} ,$ are the relaxation of water in GM, WM and CSF, respectively, with the calculation detailed below

$R_{M}$ is the relaxation of the metabolite of interest, the calculation is detailed below

The section highlighted in grey is calculated by LCModel in its metabolite water referencing, so when using the water referenced LCModel output, the equation simplifies to:

$$\left[ M \right]_{\mathrm{molal}}=\frac{(f_{\mathrm{GM}}\times R_{H2O\_GM}+ f_{\mathrm{WM}}\times R_{H2O\_WM} + f_{\mathrm{CSF}}\times R_{H2O\_CSF})}{\left( 1-f_{\mathrm{CSF}} \right) \times R_{M}}\times WaterLCMvalue$$

For tissue (GM, WM, & CSF) relaxation (R_H2O_TIS_) is calculated using:

$R_{H2O\_TIS}=\exp\left( -\frac{TE}{{T2}_{H2O\_TIS}} \right)[1-exp(-\frac{TR}{{T1}_{H2O\_TIS}})$]

In which TIS denotes the tissue compartment (GM, WM, CSF)

Metabolite relaxation (Rx) is calculated using:

$R_{x}=\exp\left( -\frac{TE}{{T2}_{x}} \right)[1-exp(-\frac{TR}{{T1}_{x}})$]

Where X denotes the metabolite, ${T2}_{x}$ is the T2 value for the metabolite, ${T1}_{x}$ is the T1 value for the metabolite, see Supplementary Table 1 for values).

For each tissue compartment (GM, WM, CSF), the mole tissue fraction is calculated.

$$f_{\mathrm{TIS}}=\frac{f_{TIS\_vol} \times d_{\mathrm{TIS}}}{f_{GM\_vol} \times d_{\mathrm{GM}}+ f_{WM\_vol} \times d_{\mathrm{WM}}+ f_{CSF\_vol} \times d_{\mathrm{CSF}}}$$

Where TIS is the tissue of interested (GM, WM, CSF) and fTIS_vol, (fGM_vol, fWM_vol and fCSF_vol) are the proportion of tissue within the voxel (taken from Gannet CoReg Stand Alone).

*d* values are the density of water within that compartment (GM, WM, CSF), taken from Supplementary Table 2.

LCModel Settings

atth2o = 0.65 (as 35 ms TE)

ATTH2O is calculated using exp(-TE/T2) as stated in the LCModel manual. As Ernst (Ernst et al., 1993) calculated the T2 of water as approximately 80ms, for this study, ATTH2O was calculated using exp(-35/80)=0.6456, rounded to 0.65.

Wconc was set to 55510 (the concentration of water) in the LCModel processing and so did not need to be corrected from the LCModel output

Metabolite Values

Supplementary Table 1 – metabolite T1 and T2 values and sources

T1 and T2 values used are the average of the GM and WM values.

| Metabolite | T1 value | Source | T2 value | Source |
| --- | --- | --- | --- | --- |
| tNAA | 1.515 | GM = 1.47  WM = 1.56  (Posse et al., 2007) | 0.274 | GM = 0.247  WM = 0.301  (Posse et al., 2007) |
| Choline | 1.23 | GM = 1.25  WM = 1.21  (Posse et al., 2007) | 0.222 | GM = 0.222  WM = 0.222  (Posse et al., 2007) |
| *myo*-inositol | 1.04 | GM = 1.12  WM = 0.96  (Posse et al., 2007) | 0.200 | GM = 0.200  WM = 0.200  (Posse et al., 2007) |
| Glutamate | 1.22 | GM = 1.22  WM = 1.17  (Mlynarik et al., 2001) | 0.135 | pACC value = 0.135  (Wyss et al., 2018) |
| Glx | 1.23 | GM = 1.25  WM = 1.21  (Posse et al., 2007) | 0.2 | GM = 0.200  WM = 0.200  (Posse et al., 2007) |
| Creatine | 1.365 | GM = 1.33  WM = 1.40  (Posse et al., 2007) | 0.17 | GM = 0.162  WM = 0.178  (Posse et al., 2007) |

Water Values

Supplementary table 2 – values used for water T1, T2 and density

| Compartment | T1 value  (Gasparovic et al., 2006) | T2 value  (Gasparovic et al., 2006) | Density  (Ernst et al., 1993) from (Gasparovic et al., 2006) |
| --- | --- | --- | --- |
| GM | 1.1304 | 0.11 | 0.78 |
| WM | 0.83 | 0.08 | 0.65 |
| CSF | 4.00 | 2.55 | 0.97 |

**References**

Ernst, T., Kreis, R., & Ross, B. D. (1993). Absolute Quantitation of Water and Metabolites in the Human Brain. I. Compartments and Water. *Journal of Magnetic Resonance, Series B*, *102*(1), 1-8. <https://doi.org/https://doi.org/10.1006/jmrb.1993.1055>

Gasparovic, C., Chen, H., & Mullins, P. G. (2018). Errors in 1H‐MRS estimates of brain metabolite concentrations caused by failing to take into account tissue‐specific signal relaxation. *NMR in Biomedicine*, *31*(6), e3914. <https://onlinelibrary.wiley.com/doi/pdf/10.1002/nbm.3914>

Gasparovic, C., Song, T., Devier, D., Bockholt, H. J., Caprihan, A., Mullins, P. G., Posse, S., Jung, R. E., & Morrison, L. A. (2006). Use of tissue water as a concentration reference for proton spectroscopic imaging. *Magn Reson Med*, *55*(6), 1219-1226. <https://doi.org/10.1002/mrm.20901>

Mlynarik, V., Gruber, S., & Moser, E. (2001). Proton T (1) and T (2) relaxation times of human brain metabolites at 3 Tesla. *NMR Biomed*, *14*(5), 325-331. <https://doi.org/10.1002/nbm.713>

Posse, S., Otazo, R., Caprihan, A., Bustillo, J., Chen, H., Henry, P. G., Marjanska, M., Gasparovic, C., Zuo, C., Magnotta, V., Mueller, B., Mullins, P., Renshaw, P., Ugurbil, K., Lim, K. O., & Alger, J. R. (2007). Proton echo-planar spectroscopic imaging of J-coupled resonances in human brain at 3 and 4 Tesla. *Magn Reson Med*, *58*(2), 236-244. <https://doi.org/10.1002/mrm.21287>

Wyss, P. O., Bianchini, C., Scheidegger, M., Giapitzakis, I. A., Hock, A., Fuchs, A., & Henning, A. (2018). In vivo estimation of transverse relaxation time constant (T2) of 17 human brain metabolites at 3T. *Magn Reson Med*, *80*(2), 452-461. <https://doi.org/10.1002/mrm.27067>
